# Supplementary material for: Behavior can be decoded across the cortex when individual differences are considered
Source: Imaging Neurosci (Camb). 2024 Nov 8;2:imag-2-00359. doi: 10.1162/imag_a_00359 (PMC12315762; doi:10.1162/imag_a_00359)
Supplement: Supplementary Material [file imag_a_00359-supp.pdf]

<Normal><Normal>**Supplemental Information**

Behavior can be decoded from across the cortex when individual differences are considered

Johan Nakuci[1*], Jiwon Yeon[2,3], Ji-Hyun Kim[4], Sung-Phil Kim[4] and Dobromir Rahnev[1]

*Corresponding author. Email: jnakuci@gmail.com

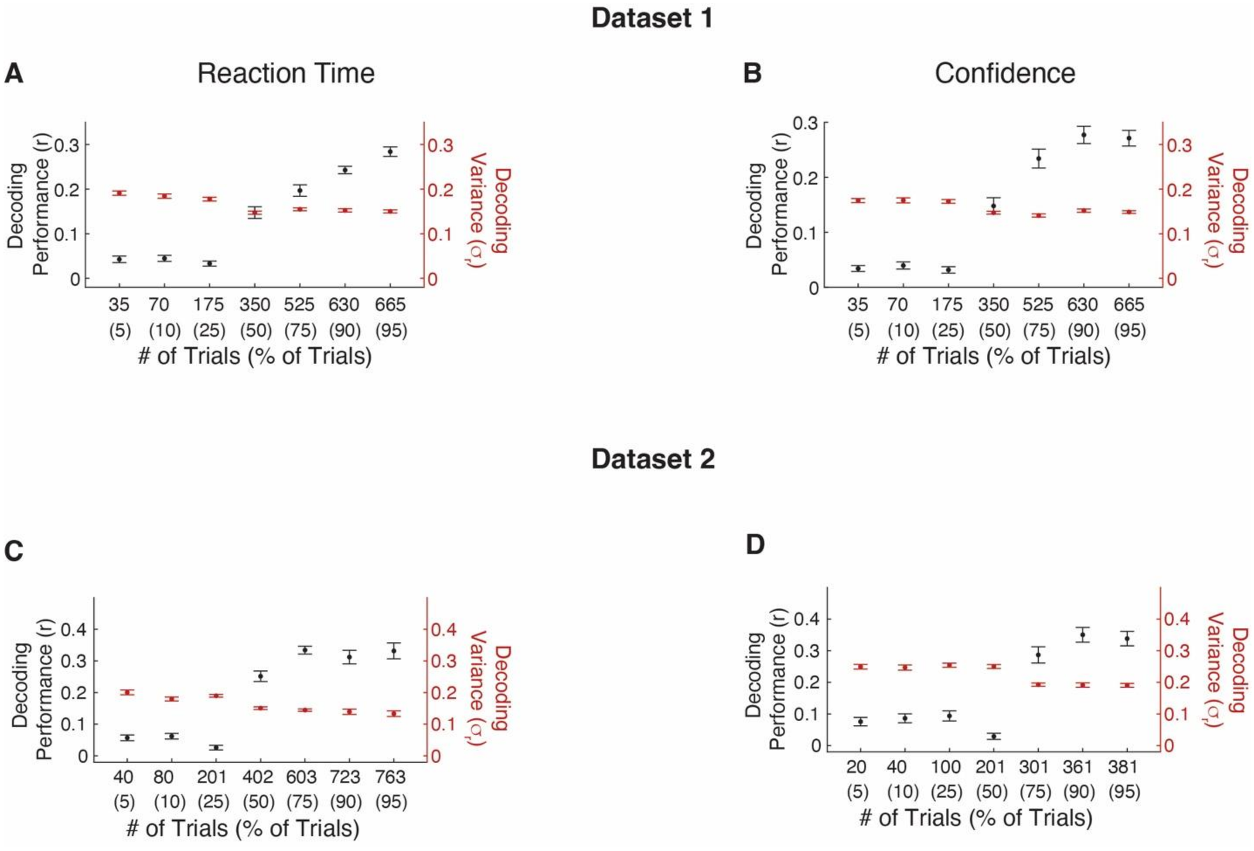

Figure S1. Robust decoding of RT and confidence requires several hundred trials when the number of trials in the test bin is fixed. In Dataset 1, the variance in decoding performance was minimized when using 350 trials (corresponding to 50% of all trials) for both (A) RT and (B) confidence. The black dots present the average decoding performance across subject after 25 repeats for each subject (*left axis)*. The red dots present the average subject-level decoding variance across the 25 repeats for each subject (*right axis)*. The number of test trial is fixed to 5% of the total trials. Error bars show SEM. (C-D) Same as Panels A-B but for Dataset 2. Note that in Dataset 2, confidence was measured on only have the trials (402) and correspondingly the decoding variance was minimized for a higher percentage of trials (75%).

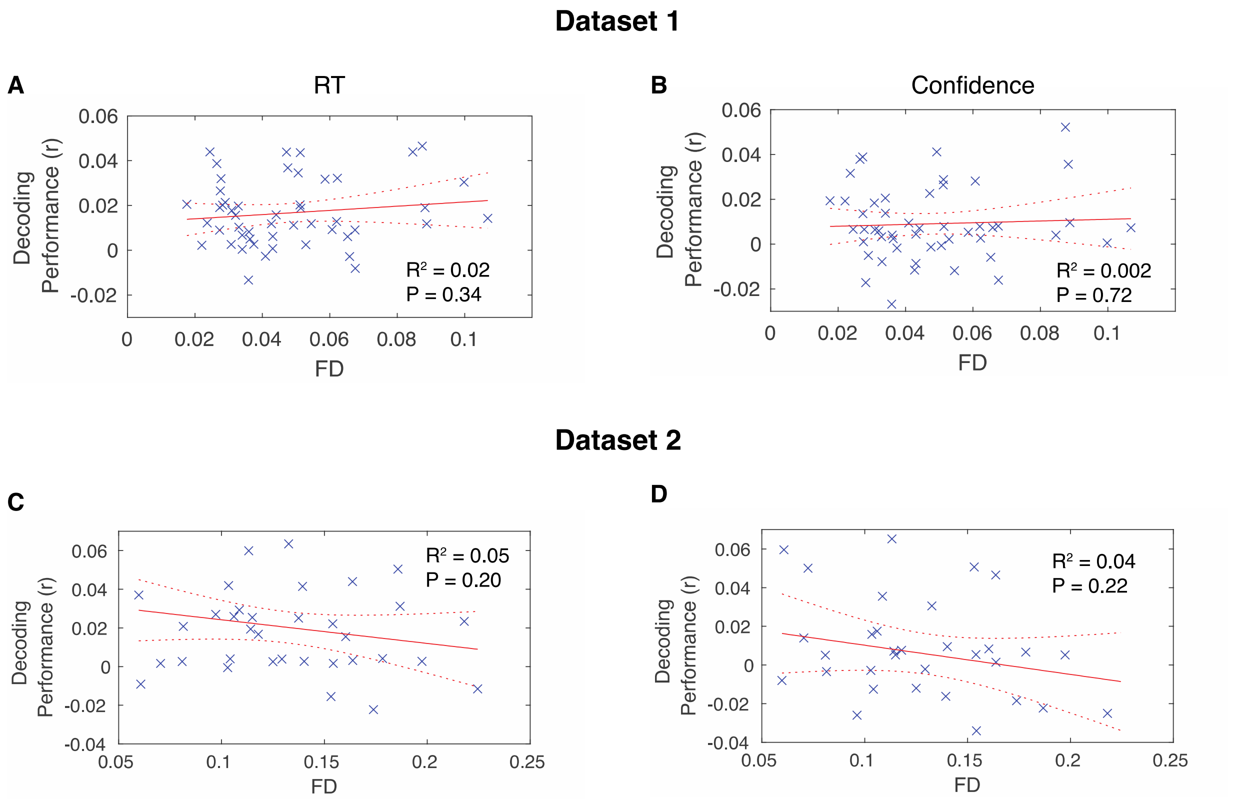

<Normal>**Figure S2. No relationship between decoding performance and frame displacement.** No significant ($P < 0.05$) association exists between decoding performance and frame displacement (FD) in both Dataset 1 (A, B) and Dataset 2 (C, D) for both RT and confidence. </Normal>
